# Supplementary material for: SAINT: A Phase I/Expanded Phase II Study Using Safe Amounts of Ipilimumab, Nivolumab and Trabectedin as First-Line Treatment of Advanced Soft Tissue Sarcoma
Source: Cancers (Basel). 2023 Jan 31;15(3):906. doi: 10.3390/cancers15030906 (PMC9913367; doi:10.3390/cancers15030906)
Supplement: Supplementary file 1 [file cancers-15-00906-s001.zip › cancers-2172607-supplementary.pdf]

Supplementary Materials

Table S1. Grade 3 or Greater Adverse Events Unrelated to Study Therapy.

**PHASE 1 – DOSE LEVEL 1 (n=3)**		
**Adverse Event**	**Grade**	
	**3**	**4**
**Gastrointestinal disorders**		
Abdominal pain	1 (33.3%)	
**PHASE 1 – DOSE LEVEL 2 (n=6)**		
**Adverse Event**	**Grade**	
	**3**	**4**
**Cardiac Disorders**		
Pericardial effusion	1 (16.7%)	
**Investigations**		
Alanine aminotransferase increased	1 (16.7%)	
**Metabolism and nutrition disorders**		
Hyponatremia	1 (16.7%)	
Hypoalbuminemia	1 (16.7%)	
**Respiratory, thoracic and mediastinal disorders**		
Pleural effusion	1 (16.7%)	
Dyspnea	1 (16.7%)	
Acute respiratory failure	1 (16.7%)	
**EXPANDED PHASE 2 (n=92)**		
**Adverse Event**	**Grade**	
	**3**	**4**
**Blood and lymphatic disorders**		
Anemia	1 (1.1%)	
**Cardiac Disorders**		
Aortic stenosis	1 (1.1%)	
Chest pain – cardiac	1 (1.1%)	
Acute coronary syndrome	1 (1.1%)	
Atrial fibrillation with RVR	2 (2.2%)	

**Endocrine disorders**		
Adrenal insufficiency	1 (1.1%)	
**Gastrointestinal disorders**		
Diarrhea	1 (1.1%)	
Anorexia	1 (1.1%)	
Nausea	1 (1.1%)	
Abdominal pain	3 (3.3%)	
Vomiting	1 (1.1%)	
RUQ pain	2 (2.2%)	
Colitis	1 (1.1%)	
**General disorders and administration site conditions**		
Fatigue	2 (2.2%)	
Edema	2 (2.2%)	
Multiorgan failure	1 (1.1%)	
Non-cardiac chest pain	1 (1.1%)	
Fluid overload	1 (1.1%)	
**Hepatobiliary disorders**		
Hepatitis, acute	1 (1.1%)	
**Infections and infestations**		
Sepsis	5 (5.4%)	
Bacteremia	1 (1.1%)	
Cellulitis	1 (1.1%)	
Urinary tract infection	2 (2.2%)	
Urosepsis	1 (1.1%)	
**Investigations**		
CPK increased	2 (2.2%)	
Prolonged QTc	1 (1.1%)	
White blood cell decreased		1 (1.1%)
CHF (dec EF 15%)		1 (1.1%)
Creatinine increased	1 (1.1%)	
Aspartate aminotransferase increased		1 (1.1%)
Alanine aminotransferase increased	1 (1.1%)	

Lymphocyte count decreased	1 (1.1%)	
**Metabolism and nutrition disorders**		
Hypokalemia	1 (1.1%)	
Hyperglycemia	3 (3.3%)	
Hypoalbuminemia	3 (3.3%)	
Hyponatremia	6 (6.5%)	1 (1.1%)
Hypocalcemia	4 (4.3%)	2 (2.2%)
Dehydration	3 (3.3%)	
Hypophosphatemia	2 (2.2%)	
Hyperbilirubinemia	1 (1.1%)	
Hyperkalemia		1 (1.1%)
**Musculoskeletal and connective tissue disorders**		
Back pain	4 (4.3%)	
Thigh pain	1 (1.1%)	
Hip pain	1 (1.1%)	
Chest wall pain	2 (2.2%)	
Neck pain	1 (1.1%)	
Arm pain	1 (1.1%)	
Hand pain	1 (1.1%)	
Flank pain	1 (1.1%)	
Shoulder pain	1 (1.1%)	
Ankle pain	4 (4.3%)	
Body pain	2 (2.2%)	
Asthenia	1 (1.1%)	
Generalized weakness	2 (2.2%)	
Pelvic pain	1 (1.1%)	
**Nervous system disorders**		
Leptomeningeal carcinomatosis	1 (1.1%)	
Altered mental status	1 (1.1%)	
Left sided sciatica	1 (1.1%)	
Nerve pain	1 (1.1%)	
**Renal and urinary disorders**		

Acute renal failure	**1 (1.1%)**	
**Respiratory, thoracic and mediastinal disorders**		
Dyspnea	7 (7.6%)	
Pleuritic chest pain	1 (1.1%)	
Hypoxic ARDS	1 (1.1%)	
Epistaxis	1 (1.1%)	
Respiratory depression	1 (1.1%)	
Cheyne stoke breathing	1 (1.1%)	
Pleural effusion	1 (1.1%)	
Acute hypoxic respiratory failure	1 (1.1%)	
PNA, community acquired	1 (1.1%)	
Pneumothorax	1 (1.1%)	
Hypoxia	2 (2.2%)	
Pneumonia	1 (1.1%)	
Pulmonary embolism	1 (1.1%)	
**Vascular disorders**		
Hypertension	1 (1.1%)	
Acute thrombus right subclavian	1 (1.1%)	

Table S2. Listing of subtypes, mutation, no. of cycles, BORR, PFS and OS.

Patient	Sarcoma Histology	Mutation(s), if known	Number of Cycles Treated	Best Response RECIST v1.1	Best Response iRecist	PFS, weeks	OS, weeks
**PHASE I-DOSE LEVEL I**							
1	Leiomyosarcoma	none	4	SD	SD	35	203
2	Liposarcoma, dedifferentiated	pan-TRK expression	<1	SD	SD	6	13
3	Leiomyosarcoma	none	2	SD	SD	25	85
**PHASE I-DOSE LEVEL II**							
4	Desmoplastic small cell tumor with myogenic/rhabdomyoblastic differentiation	EWSR1-WT1 fusion, TMB-Low	20	SD	SD	67	126
5	Rhabdomyosarcoma		<1	NE	NE	NE	15
6	Osteosarcoma	none	1	PD	PD	14	24

7	Leiomyosarcoma	none	3	SD	SD	19	184
8	Liposarcoma, de-differentiated	p53 WILDTYPE MDM2 amplification	14	SD	SD	49	95
9	Rhabdomyosarcoma	none	5	SD	SD	28	36
**EXPANDED PHASE II**							
1	Leiomyosarcoma	none	6	SD	SD	21	74
2	Liposarcoma, de-differentiated	CDK4, MDM2, ESR1 amplification, MLL2 alteration	13	SD	SD	78	202
3	Round cell sarcoma	CIC-DUX4 fusion	<1	NE	NE	NE	13
4	Myxoid chon-drosarcoma	GNAS R201C; MLH1: Positive 2+,	13	SD	SD	72	200

		90%; MSH2: Positive 2+, 90%; MSH6: Positive 1+, 90%; PMS2: Positive 1+, 90%					
5	Pleomorphic sarcoma	none	2	SD	SD	19	19
6	Synovial sarcoma	none	7	SD	SD	44	97
7	Leiomyosarcoma	TMB - 2 Muts/Mb, MAP3K6 - Splice site 1256-2A>G, RB1 - Loss, TP53 - Loss	11	PR	PR	86	199
8	Undifferentiated pleomorphic sarcoma	none	4	SD	SD	27	32

9	Myxoid round cell liposar-coma	NYESO positive tumor; MLH1 - Positive 2+, 90%; MSH2 - Positive 3+, 90%; MSH6 - Positive 2+, 80%; PMS2 - Positive 2+, 80%	12	PR	PR	92	137
10	Leiomyosarcoma	EGFR, MET(+)	12	SD	SD	53	197
11	Pleomorphic liposarcoma	c-KIT Exon 11 mutation (p. V560del)	<1	SD	SD	63	79
12	Pleomorphic sarcoma	EGFR (amplification), RICTOR (amplification), CRKL	10	CR (surgical)	CR (surgical)	59	196

		(amplification), EPHA3 (amplification), FGF10 (amplification), MALT1 (amplification), MAPK1 (amplification), RB1 (loss), TP53 (loss); MLH1 - Positive 1+, 80%; MSH2 - Positive 2+, 95%; MSH6 - Positive 2+, 95%; PMS2 - Positive 1+, 90%					
13	Alveolar soft part sarcoma	none	12	SD	SD	43	92

14	Undifferentiated pleomorphic sarcoma	none	15	CR	CR	105	191
15	Osteosarcoma	none	2	SD	SD	4	49
16	Leiomyosarcoma	Pathogenic Variant: ATRX, TP53; ER: Positive 1+, 90%; MLH1: Positive 1+, 100%; MSH2: Positive 2+, 95%; MSH6: Positive 1+, 10%; PMS2: Positive 1+, 40%; PR: Positive 1+, 90%; PTEN: Positive 2+, 85%	11	SD	SD	42	157
17	Leiomyosarcoma	none	1	NE	NE	NE	11

18	Leiomyosarcoma	none	2	SD	SD	6	7
19	Peripheral nerve sheath tumor	BRAF, PTEN, MYC, ARID2, CDKN2A, CHEK2, EPHA7, LRP1B, ROS1 (+)	<1	NE	NE	NE	31
20	Pleomorphic rhabdomyosarcoma	none	1	PD	PD	15	22
21	Pleomorphic rhabdomyosarcoma	none	<1	NE	NE	NE	4
22	Clear cell sarcoma	EWSR1-ATF1 Fusion	4	PR	PR	30	52
23	Epithelioid sarcoma	NYESO Positive 2%	2	SD	SD	14	88

24	Angiosarcoma	Pathogenic Variant: POT1; MLH1: Positive 3+, 95%; MSH2: Positive 3+, 95%; MSH6: Positive 2+, 95%; PMS2: Positive 2+, 95%	2	PD	PD	6	169
25	Liposarcoma, de-differentiated	CDK4 - Amplified; MSI-Intermediate; AKT2 - Amplified; CD274 (PD-L1) - Amplified; MDM2 - Amplified ; MLH1 -	8	SD	SD	32	167

		Positive 1+, 25%; MSH2 - Positive 1+, 100%; MSH6 - Positive 1+, 75%; PMS2 - Positive 1+, 75%					
26	NOS Sarcoma	ER/PR (+), HER2 (--)	4	SD	SD	13	55
27	Clear cell sarcoma	Foundation One: EWSR1-ATF1 fusion, Tumor Mutation Burden Low	1	NE	NE	NE	5
28	Clear cell sarcoma	Paradigm: SETD2 (+); MLH1, MSH2, MSH6, PMS2, hENT1 (+)	9	SD	SD	28	37
29	Undifferentiated pleomorphic sarcoma	none	8	CR (surgical)	CR (surgical)	50	163

30	Solitary fibrous tumor	none	6	SD	SD	20	162
31	Synovial sarcoma	none	7	CR	CR	56	140
32	Myxoid liposarcoma	FGFR2(+); CDK4 (+); FLT4 (+); MDM2 (+); RICTOR (+); RNF43 (+)	9	SD	SD	27	107
33	Myxoid liposarcoma	MLH1, MSH2, MSH6, PMS2 (+)	10	SD	SD	30	158
34	Synovial sarcoma	CTNNB1 (+), MSH2 (+)	7	PR	PR	22	48
35	Myxofibrosarcoma	none	6	SD	SD	19	37
	and giant cell tumor components						
36	Rhabdomyosarcoma	none	10	SD	SD	34	156
37	Carcinosarcoma	none	12	PD	PD	6	18

38	Leiomyosarcoma	PTEN, Microsatellite-stable, PC, TP53, TMB-low	8	SD	SD	53	134
39	Liposarcoma	CDK4 amplification, MDM2 amplification	6	SD	SD	18	99
40	Leiomyosarcoma	none	6	CR	CR	23	146
41	Leiomyosarcoma	ALK TNS1-ALK fusion and SMAR-CAL1-ALK fusion;	2	PD	PD	4	146
		CDKN2A/B loss; MED12 G44A; RUNX1 V130fs*8					
42	Leiomyosarcoma	ATRX (+); TP53(+); CAIX (+); TOPO1(+); MGMT(+)	<1	NE	NE	NE	9

43	Myxofibrosarcoma	none	1	NE	NE	NE	4
44	Myxofibrosarcoma	PDL1 (+), APC (+), TP53 (+)	10	CR	CR	32	143
45	Peripheral nerve sheath tumor	none	<1	NE	NE	NE	8
46	Liposarcoma	TMB Intermediate (12 mutations/Mb)	7	PR	PR	26	84
47	Solitary fibrous tumor	CREBBP (+)	8	SD	SD	40	81
48	Undifferentiated pleomorphic sarcoma	PTEN (+); NTRK2 (+)	1	SD	SD	15	54
49	Peripheral nerve sheath tumor	NF1 (+)	16	SD	SD	79	125

50	Stromal sarcoma	CREBBP: Mutated, Pathogenic; IDH2: Mutated, Pathogenic; MED12: Mutated, Pathogenic; PRDM1: Mutated, Path-ogenic; MLH1 Positive 1+, 100%; MSH2 Positive 3+, 100%; MSH6 Positive	6	PR	PR	20	61
		1+, 100%; PMS2 Positive 1+, 100%; PTEN Positive 2+, 100%					

51	Sarcoma with myoid differentiation	none	3	SD	SD	15	17
52	Liposarcoma	CDK4 (+); RB1(+); CD274 (PD-L1), MDM2, TOP1 - Amplified	3	SD	SD	14	70
53	Desmoplastic small round cell	EWSR1-WT1 fusion	9	PR	PR	39	122
54	Liposarcoma	CDK4 (+), MDM2 (+), PD-L1 (+) - 5%	10	CR	CR	31	118

55	Leiomyosarcoma	MLH1 Positive 1+, 75%; MSH2 Positive 2+, 100%; MSH6 Positive 2+, 65%; PMS2 Positive 1+, 10%, PTEN Pathogenic Variant Exon 5 p.D92E; RB1 Pathogenic Variant Exon 17 c.1695+1G>T; TP53 Pathogenic Variant Exon 6 p.E224	8	PR	PR	30	117
56	Synovial sarcoma	none	9	PR	PR	33	34
57	Stromal sarcoma	ER (+), PR (+), TMB - intermediate, PTEN positive	2	PD	PD	9	54

58	Undifferentiated pleomorphic sarcoma	PD-L1 - 5%	2	SD	SD	15	64
59	NOS sarcoma	EWSR1-CREB3L2 fusion	8	SD	SD	25	109
60	Myxofibrosarcoma	none	9	PR	PR	49	52
61	Leiomyosarcoma	TMB - Intermediate, ATRX: Pathogenic Variant Exon 9 p.R781; AURKB: Amplified	16	SD	SD	65	108
62	Undifferentiated	PD-L1 TPS 5%, PTEN loss, CKS1B	2	PD	PD	7	107

	pleomorphic sarcoma	amplification, MCL1 amplification, RB1 loss, TOP1 amplification, TP53 mutation, TSC 2+ marker present					
63	Carcinosarcoma	TMB - intermediate; PTEN: positive, 90%; KRAS - G12D; MUTYH: pathogenic variant; TP53 (+)	6	SD	SD	19	51
64	Pleomorphic sarcoma	ATRX - Pathogenic Variant Exon 9 p.T363fs; TP53	11	SD	SD	32	73

		- Pathogenic Variant Exon 5 p.H168R					
65	Undifferentiated pleomorphic sarcoma	NF1 (+); PK3CA (+); PTEN (+); TP53 (+); PD-L1 (+) - 30%	6	SD	SD	20	104
66	Synovial sarcoma	none	6	SD	SD	22	103
67	Leiomyosarcoma	TMB - intermediate; ATRX (+), RET (+)	1	PD	PD	7	17
68	Spindle cell sarcoma	RB1 - loss; TP53 - loss; HGB, IKZF3, MLL2, MSH3, MYST3, NSD1, PDG-FRB, RAD50, TET2, TUSC3,	8	CR surgical	CR surgical	30	94

		WDR90 (+); MLH1: Positive 2+, 60%; MSH2: Positive 2+, 85%; MSH6: Positive 2+, 85%; PMS2: Positive 2+, 85%					
69	Giant Cell Tumor	PD-L1 (+) - 5%; KRAS (+) -G12F; PIK3R1 (+); POT1 (+)	1	SD	SD	4	26
70	Leiomyosarcoma	NTRK1 Fusion Detected -- Exon 10 CIK4A2-NTRK1; TP53 (+); PTEN (+); MTAP - loss;	2	NE	NE	NE	22

		KIT - amplification; CDKN2A - loss; CDKN2B - loss; NF1 - loss; EGFR amplification; MEN1 - loss; KDR amplification; PDGFRA - amplification; TP53 - E221*					
71	Clear cell sarcoma	none	1	NE	NE	NE	6
72	Liposarcoma	MLH1 - Positive 2+, 98%; MSH2 - Positive 3+, 95%; MSH6 - Positive 1+, 99%; PMS2 - Positive 1+, 99%	13	SD	SD	48	84

73	Leiomyosarcoma	CDKN2A, CDKN2B deep deletion; CD10 (+); pan-TRK (+); ZC3H7B: Pathogenic Fusion Detected; MLH1 Positive 2+, 95%; MSH2 Positive 2+, 95%; MSH6 Positive 1+, 20%; PMS2 Positive 2+, 95%; PTEN Positive 2+, 100%	3	SD	SD	12	84
74	Liposarcoma	CDK4 (+); MDM2 (+);	<1	NE	NE	NE	13
		JUN - amplification					

75	Pleomorphic sarcoma	PTEN - Alteration: Loss; TP53 - Alteration: V216L; PTEN - PTEN-CFL1P1 rearrangement; KDR - L462V; RAF1 - N553H; FGFR3 - P572L; TSC1 - R37H	5	SD	SD	16	34
76	Liposarcoma	PTEN - loss; JUN - amplification; ATR - splice site 5739-1G>A; RB1 -	12	SD	SD	41	81

		E466*; ATRX - splice site 6849+2T>C; Tumor Mutational Burden - 8.83 mutations-per-megabase					
77	Leiomyosarcoma	PTEN - loss; MED12 - G44D; TP53 - Y236C; Tumor Mutational Burden - 2.52 mutations-per-megabase; MED12 - Pathogenic Variant Exon 2 p.Y236C; TP53 - Pathogenic	13	SD	SD	43	63
		Variant Exon 7 p.Y236C					

78	Undifferentiated pleomorphic sarcoma	PD-L1 28-8 POSITIVE; MSI - indeterminate; TMB - indeterminate; NTRK1/2/3 - indeterminate	5	SD	SD	20	75
79	Spindle cell sarcoma	NF1 - Pathogenic Variant Exon 1/ | p.G629R; SETD2 - Pathogenic Var- iant Exon 3 - (Histone- ly- sine N- methyl- transfera se in- hibitors) ; MLH1 Positive	2	PD	PD	6	27

		2+, 90%; MSH2 Positive 3+, 95%; MSH6 Positive 1+, 100%; PMS2 Positive 1+, 100%					
80	Leiomyosarcoma	none	<1	NE	NE	NE	3
81	Uterine sarcoma	CDK4: amplification; MDM2: amplification; ATM: V734fs*2; Tumor Muta-tional Burden: 1.26 muta-tions-per-megabase; MAP2K4: amplification; BCORL1:	3	SD	SD	12	67

		P467A; ERBB3: G780E; CARD11: S694L; AXL: V150A; FANCG: A153G; HSD3B1: L212R; CHEK2: D82_E86del					
82	Undifferentiated pleomorphic sarcoma	PD-L1 (SP142): Positive 2+, 5%; MLH1: Positive 2+, 95%; MSH2: Positive 2+, 95%; MSH6: Positive 1+, 95%; PMS2:	<1	PD	PD	6	66
		Positive 3+, 80%					

83	Leiomyosarcoma	ER positive 2+, 100%; MSH6 Positive 1+, 60%; ATRX Pathogenic Variant Exon 9 p.K690fs; CHEK2 Likely Pathogenic Variant Exon 4 p.I157T	2	PD	PD	8	64
84	Leiomyosarcoma	PR - Positive 1+, 10%; MED12 - Pathogenic Variant Exon 2 p.G44C, MSH2 Positive 2+, 80%; MSH6	9	SD	SD	29	41

		Positive 2+, 50%; PTEN Positive 2+, 100; MLH1 Positive 1+, 40%; PMS2 Positive 2+, 95%					
85	Liposarcoma	MDM2: Amplification; ZNF217: Amplification; CDK4: Amplification ; PIK3C2G: Amplification; ATRX: Loss; MUTYH - R505W; WT1 - A100G; TSC1 - K587R; CDK4: Amplified;	1	NE	NE	5	8
		MDM2: Amplified					

86	Leiomyosarcoma	C17orf39 amplification, ESR1 amplification; RB1 loss; PTEN loss; AKT1 amplification; ATRX ATRX-BAGE4;BAGE5;BAGE2;BAGE3 truncation; PIK3CA E545K; TP53 P390fs*32; TP53 Pathogenic Variant, Exon 11 p.P390fs; PIK3CA	11	CR	CR	34	59

		-Pathogenic Variant Exon 10 p.E545K; ER Positive 2+, 30%; MLH1 -Positive 2+, 30%; MSH2 -Positive 2+, 30%; MSH6 -Positive 2+, 35% ; PMS2 - Positive 2+, 35%; PTEN - Positive 2+, 100%					
87	Leiomyosarcoma	MAP3K6 splice site 1256-2A>G; RB1 R445*; TP53 loss exons 2-4	17	PR	PR	55	59

88	Leiomyosarcoma	RB1 - loss; PTCH1 - splice site 2561-2A>G; ATRX - E723fs*9; TP53-R209fs*6; TMB - 1.26 mutations-per-megabase; ATRX - Pathogenic; Variant, Exon 9 p.E723fs; PTCH1 - Pathogenic Variant, Exon 16 c.2561-2A>G; TP53 - Pathogenic Variant, Exon 6 p.R209fs; PD-L1 (SP142) -	6	PR	PR	29	54

		Positive 2+, 5%; ER - Positive 2+, 45%; MLH1 - Positive 1+, 45%; MSH2 - Positive 2+, 90%; MSH6 - Positive 2+, 90%; PMS2 - Positive 2+, 50%; PTEN - Positive 2+, 100%					
89	Leiomyosarcoma	ER Positive; MLH1 Positive 1+, 90%; MSH2 Positive 2+, 95%; MSH6 Positive 2+, 95%; PTEN Positive 1+, 100%;	8	SD	SD	27	56
		PMS2 Positive 2+, 90%					

90	Rhabdomyosarcoma	PIK3CA p.H1047L; NM_006218.2: c.3140A>T; Estimated variant allele frequency: 31%	5	SD	SD	18	54
91	Liposarcoma, dedifferentiated	MTAP loss; ATRX loss; FGFR1 amplification; CDKN2B loss; CDKN2A loss; BCL2 amplification; EPHB1 amplification; MDM2 amplification; CDK4 mplification; CHEK2 T367fs*15	<1	SD	SD	10	10

92	Liposarcoma	CDK4 amplification; MDM2 amplification; MTAP amplification; CDK4 CDK4-OS9 rearrangement; FGFR3 P129L	7	SD	SD	24	49
